# Concurrent Prediction of Successful Aging by Objective Markers of Aging and Psychological Markers of Aging

**DOI:** 10.1093/geroni/igaf122.4089

**Published:** 2025-12-31

**Authors:** Fabio Selovin, M Clara de Paula Couto, Markus Scholz, Klaus Rothermund

**Affiliations:** Friedrich-Schiller Universität Jena, Institut für Psychologie, Jena, Thuringen, Germany; Friedrich-Schiller-University Jena, Jena, Thuringen, Germany; University of Leipzig, Leipzig, Sachsen, Germany; Friedrich-Schiller University Jena, Institute of Psychology, Jena, Thuringen, Germany

## Abstract

Objective indicators of cognitive decline, immune function and inflammation are used as objective markers of aging (OMAs) to predict an individuals aging success. Individuals beliefs and evaluations of their own age and aging, like subjective age have been found to significantly impact longevity and health in old age, making them psychological markers of aging (PMAs). However, studies rarely consider PMAs alongside OMAs when predicting successful aging related outcomes. Our study aims to concurrently predict autonomous daily functioning (IADL), health-related quality of life (hQoL) and life satisfaction (LS) using OMAs of cognitive aging (trail making test), inflammation (interleukin-6, c-reactive protein), and immune aging (neutrophil-to-lymphocyte ratio) as well as PMAs, specifically subjective age and future self-views. To this end, we used cross-sectional data from 456 older adults (Md age = 74 years, 60-86 years) from the LIFE Adult Study and predicted the respective outcome by hierarchical linear regression, while controlling for socio-demographic factors. The results show that OMAs of cognitive function predicted IADL (ß = -0.15, p < .05) and OMAs of inflammation predicted hQoL (ß = -0.10, p < .05) but no OMA significantly predicted LS. Subjective age (ß = -0.17, p < .05) and future self-views (ß = 0.15, p < .05) predicted LS, but did not predict IADL. Future self-views (ß = 0.27, p < .001) but not subjective age was associated with hQoL. Our results suggest that fundamental biological aging pathways and psychological constructions of age are selectively associated with specific form of success in old age.

